# SHARE – workpackage 5: evidence-based recommendations for diagnosis and treatment of rare paediatric vasculitides

**DOI:** 10.1186/1546-0096-12-S1-P121

**Published:** 2014-09-17

**Authors:** Nienke de Graeff, Noortje Groot, Sylvia Kamphuis, Pavla Dolezalova, Despina Eleftheriou, Annet van Royen, Marinka Twilt, Seza Ozen, Paul Brogan, Michael Beresford

**Affiliations:** 1 Wilhelmina Children's Hospital, Utrecht, Netherlands; 2 Alder Hey Children's Hospital, Liverpool, UK; 3 Sophia Children's Hospital, Erasmus Medical Centre, Rotterdam, Netherlands; 4 General University Hospital, Prague, Czech Republic; 5 Great Ormond Street Hospital for Children, London, UK; 6 Aarhus University Hospital, Aarhus, Denmark; 7 Dept. of Pediatric Rheumatology, Hacettepe University, Ankara, Turkey; 8 University of Liverpool, Liverpool, UK

## Introduction

Polyarteritis Nodosa (PAN), Granulomatosis with Polyangiitis (GPA), Microscopic Polyangiitis (MPA), Eosinophilic Granulomatosis with Polyangiitis (EGPA) and Takayasu Arteritis (TA) are rare paediatric vasculitides that can lead to significant morbidity. Evidence-based guidelines are sparse and management is mostly based on physician experience. Consequently, treatment regimens differ throughout Europe. In 2012, a European initiative called SHARE (Single Hub and Access point for paediatric Rheumatology in Europe) was launched to optimize and disseminate guidelines for diagnosis and management for children and young adults with paediatric rheumatic diseases (PRD) such as vasculitis within Europe.

## Objectives

To provide evidence-based recommendations for diagnosis and treatment of paediatric vasculitides, specifically PAN, GPA, MPA, EGPA and TA.

## Methods

Evidence based recommendations were developed using the European League Against Rheumatism (EULAR) standard operating procedure. An expert committee was formed, consisting of paediatric rheumatologists from across Europe with expertise in vasculitis. The expert committee defined search terms for the systematic literature review, which was performed in summer 2013. Two independent experts scored each article for validity and level of evidence. Recommendations derived from the literature were evaluated using an online survey. Those with less than 80% agreement during the online survey were reformulated. Subsequently, all recommendations will be discussed at a consensus meeting using the nominal group technique [1]. Recommendations will be accepted if more than 80% agreement is reached.

## Results

The systematic literature search yielded 7766 articles, including articles on two more common forms of paediatric vasculitis, Kawasaki Disease (KD) and Henoch Schonlein Purpura (HSP). After exclusion of these articles and articles that did not meet inclusion criteria, 93 articles on rare paediatric vasculitides were considered relevant. The expert committee then scored these for validity and level of evidence. Evidence supporting recommendations for diagnosis and treatment was extracted from the literature. Subsequently, statements on clinical symptoms, referral of patients, useful laboratory investigations, imaging techniques and treatment were formulated based on this evidence and expert opinion and were evaluated in an online survey. The outcome of this survey will be discussed at the next consensus meeting with the aim of yielding final recommendations on minimal standards of care for children with vasculitis throughout Europe.

## Conclusion

The SHARE initiative provides recommendations for diagnosis and treatment of paediatric vasculitides and thereby facilitates improvement and uniformity of care for patients throughout Europe. Currently, similar processes are ongoing to add add guidelines on holistic care for PRD patients. As a final result, SHARE will provide standards of minimal care for different PRDs, including rare vasculitides (PAN, GPA, MPA, EGPA and TA) as well as more common vasculitides (KD and HSP).

## Disclosure of interest

None declared.
